# Inflammatory response to retrotransposons drives tumor drug resistance that can be prevented by reverse transcriptase inhibitors

**DOI:** 10.1073/pnas.2213146119

**Published:** 2022-11-30

**Authors:** Ksenia A. Novototskaya-Vlasova, Nickolay S. Neznanov, Ivan Molodtsov, Brandon M. Hall, Mairead Commane, Anatoli S. Gleiberman, Jayne Murray, Michelle Haber, Murray D. Norris, Katerina I. Leonova, Andrei V. Gudkov

**Affiliations:** ^a^Department of Cell Stress Biology, Roswell Park Comprehensive Cancer Center, Buffalo, NY 14203; ^b^Genome Protection, Inc., Buffalo, NY 14263; ^c^Children’s Cancer Institute, Lowy Cancer Research Centre, Sydney, NSW 2052, Australia; ^d^School of Clinical Medicine, UNSW Medicine & Health, University of New South Wales, Sydney 2052, NSW, Australia; ^e^University of New South Wales Centre for Childhood Cancer Research, Sydney 2052, NSW, Australia

**Keywords:** LINE-1, progression-free survival, interferon, NF-kappaB, multidrug resistance

## Abstract

Activation of endogenous retrotransposons frequently occurs in cancer cells and contributes to tumor genomic instability. To test whether inhibition of retrotranspositions has an anticancer effect, we used treatment with the nucleoside reverse transcriptase inhibitor (NRTI) stavudine (STV) in mouse cancer models, MMTV-HER2/Neu and Th-MYCN, that spontaneously develop breast cancer and neuroblastoma, respectively. In both cases, STV in drinking water did not affect tumor incidence nor demonstrate direct antitumor effects. However, STV dramatically extended progression-free survival in both models following an initial complete response to chemotherapy. To approach the mechanism underlying this phenomenon, we analyzed the effect of NRTI on the selection of treatment-resistant variants in tumor cells in culture. Cultivation of mouse breast carcinoma 4T1 in the presence of STV dramatically reduced the frequency of cells capable of surviving treatment with anticancer drugs. Global transcriptome analysis demonstrated that the acquisition of drug resistance by 4T1 cells was accompanied by an increase in the constitutive activity of interferon type I and NF-κB pathways and an elevated expression of LINE-1 elements, which are known to induce inflammatory responses via their products of reverse transcription. Treatment with NRTI reduced NF-κB activity and reverted drug resistance. Furthermore, the inducible expression of LINE-1 stimulated inflammatory response and increased the frequency of drug-resistant variants in a tumor cell population. These results indicate a mechanism by which retrotransposon desilencing can stimulate tumor cell survival during treatment and suggest reverse transcriptase inhibition as a potential therapeutic approach for targeting the development of drug-resistant cancers.

## Significance

The role of endogenous retrotransposons in cancer initiation and progression has been traditionally viewed in the context of their mutagenic activity. The main conclusion of the present work is that retrotransposons can stimulate treatment resistance by the induction of prosurvival inflammatory pathways, a mechanism that is distinct from retrotransposon-mediated mutagenesis. Furthermore, this mechanism is “druggable”, evidenced by partial reversion via reverse transcriptase inhibitors. In this regard, drugs targeting L1-expressing cells represent a category of anticancer agents that lack direct antitumor activity but are capable of suppressing tumor adaptability. This type of pharmaceutical is projected to improve the outcome of any anticancer treatment by extending progression-free survival following initial response.

Adaptive phenotypic plasticity is the most important property of cancer in determining the ability of tumor cells to overcome natural tumor-suppressive mechanisms (e.g., immune response, hypoxia, etc.) (1) and pharmacological antitumor treatments (2). Tumor cell populations typically contain a low proportion of cells capable of surviving therapies, the expansion of which results in relapses of resistant variants requiring adjustment of treatment modalities (3). Continuous tumor progression along this path eventually leads to the fatal exhaustion of treatment options (4).

Despite the growing number of approved anticancer drugs targeting tumor viability and proliferation, there are currently no approaches targeting tumor “creativity”, i.e., its extraordinary ability to evolve and adapt. In addition, there is an obvious reason for this, namely that this property of cancer has been traditionally associated with genomic instability that was attributed to decreased accuracy of DNA replication and repair, increased oxidative stress due to impaired metabolism of reactive oxygen species, and loss of intrinsic mechanisms underlying elimination of cells with damaged DNA (5) (i.e., loss of p53 function that mediates senescence and apoptosis) (6). Restoration of these control mechanisms cannot be reached by drug-based interventions, making genomic instability seemingly “undruggable”. However, recent discoveries may lead to a cardinal revision of this paradigm.

There is a growing body of evidence revealing the existence of a specific, intrinsic mechanism that frequently occurs in cancer cells and is capable of generating genomic instability: activation of endogenous retrotransposons (7). In fact, epigenetic desilencing of LINE-1 (L1) retrotransposons, the most abundant family of self-replicating virus-like interspersed repeats, occupying more than 20% of the human genome, is observed in a high proportion of human tumors (8). Intact copies of L1 encode both ORF1p, an RNA-binding protein, and ORF2p, a multifunctional protein that combines reverse transcriptase (RT) and endonuclease (integrase) activities. ORF2p, with the assistance of ORF1p, performs the reverse transcription of L1 RNA into cDNA and integrates this new L1 copy into chromosomal DNA. In addition to replicating its own genome, L1 can generate cDNA copies of RNAs transcribed from short interspersed nuclear elements (SINEs) (9), an abundant class of nonautonomous retrotransposons (represented in primates by the Alu family of repeats), as well as RNAs transcribed from pericentromeric satellite DNA consisting of tandem repeats (10). On rare occasions, L1 RT can also reverse transcribe messenger RNAs, resulting in the appearance of intron-less, promoter-less cDNA copies of protein-coding sequences known as processed pseudogenes (11). In addition to L1-generated genetic instability (i.e., via insertional mutagenesis and endonuclease-generated DNA breaks), L1 also generates epigenetic instability by creating new copies of elements that are prone to epigenetic silencing (12). Tumor suppressors p53 and Rb have been shown to be involved in the control of epigenetic silencing of retrotransposons and satellite DNA; their loss results in the activation of “retrobiome” expansion (13) and thus provides an additional mechanistic explanation for the well-known connection between the loss of these tumor suppressors and genomic instability.

The desilencing of the retrobiome frequently occurs in cancer (14), likely as a consequence of global epigenetic reprogramming in tumor cells. This retrobiome activation leads to the induction of an antiviral inflammatory response, including the cGAS-STING pathway (triggered by the presence of cytoplasmic DNA synthesized by L1) and possibly other innate immunity receptors (15). This phenomenon, which we named transcription of repeats activates interferon (TRAIN (16)), is another important reflection of retrotransposon activity that contributes not only to cancer phenotype but also to aging-related inflammation since L1 activation can also occur in senescent cells (17).

The potential consequence of L1 desilencing in tumor cells opens the opportunity for pharmacological control over the progression of genomic instability in cancer, such as through use of pharmacological inhibitors of L1 RT activity. In fact, nucleoside reverse transcriptase inhibitor (NRTI) developed to treat HIV and hepatitis B infections (such as stavudine (STV), lamivudine, tenofovir, islatravir, etc.) (18) are capable of inhibiting L1 RT (19). The principal ability of this approach, i.e., utilizing NRTI to counteract L1-driven genomic instability, was demonstrated earlier in a model of RT-mediated retrotranspositions of pericentromeric satellite DNA (20). Furthermore, NRTI treatment was also capable of suppressing L1-mediated inflammation and expansion, as demonstrated in recent work from our and other groups (21, 22). These promising results led us to test the effects of NRTI on tumor development and progression—particularly on the frequency of acquisition of treatment resistance.

Here, we describe the results of this testing conducted in two mouse cancer models, MMTV-HER2/Neu (23) and Th-MYCN (24) mice, that spontaneously develop breast cancer and neuroblastoma, respectively. In both cases, NRTI treatment was found to have nearly no effect on cancer occurrence but demonstrated a profound delay in the onset of tumor relapses, thus greatly prolonging progression-free survival. To decipher the molecular mechanisms by which retrotransposons stimulate tumor cell survival during treatment, we undertook a detailed analysis of mouse breast cancer cells by either enforcing or inhibiting their retrotransposon activity and by analyzing the role of L1 expression in acquired drug resistance. This analysis brought us to the unexpected conclusion that tumor drug resistance, reversible by NRTI, is associated with elevated, constitutive NF-κB and interferon (IFN) type I responses induced by L1 activation. We conclude that the stimulation of tumor progression by L1 does not necessarily require retrotranspositions and can be driven by activation of proinflammatory, prosurvival mechanisms.

## Results

### NRTI Does Not Affect Tumor Occurrence but Prolongs Progression-Free Survival in Mouse Cancer Models.

**Fig. 1.**
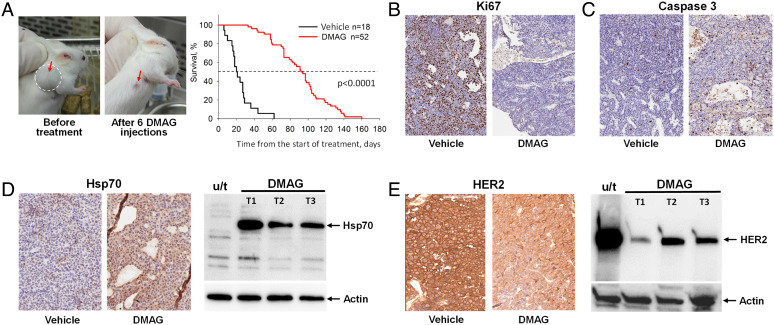
Response of mammary tumors in MMTV-HER2/Neu mice to 17-DMAG treatment. (*A*) 17-DMAG treatment (3–6 IP injections at 20 mg/kg dose) results in complete response in most tumors (as seen in representative photos) and significant extension of lifespan for tumor-bearing animals. *P* < 0.0001 for the difference in average lifespan (see dashed line in Kaplan–Meier survival plot) between untreated (20 d) and treated (100 d) mice, (Kaplan–Meier log-rank test). (*B–E*) Immunohistochemical staining of mammary tumors from mice given three IP injections of vehicle or 17-DMAG for Ki67, a marker of proliferation (*B*), activated caspase 3, a marker of apoptosis (*C*), Hsp70, an indicator of heat shock response activation (*D*), and HER2/Neu, which is regulated by Hsp90 (*E*). For *D–E*, western blot analysis of proteins in tumor extracts is provided (one tumor from an untreated (u/t) animal and three tumors from three different 17-DMAG-treated mice (T1–T3)), with actin serving as a housekeeping control.

To analyze the effect of NRTI on spontaneous tumor development and acquisition of resistance to treatment, we used MMTV-HER2/Neu mice carrying in their germline a transgenic cassette comprised of rat HER2/Neu cDNA under the control of the promoter from the long terminal repeat of mouse mammary tumor virus (MMTV) that drives tissue-specific expression of the proto-oncogene in the breast epithelium (23). 100% of MMTV-HER2/Neu females develop spontaneous breast tumors between 4 and 18 mo (23). To establish a model of acquired treatment resistance, we were interested in selecting a drug that was initially effective against primary tumors, relatively safe (thereby enabling long-term application), and allowed tumors to relapse regardless of continuous treatment. Most of the drugs tested were ruled out either due to prohibitive toxicity at efficacious doses (e.g., doxorubicin or cyclophosphamide) or insufficient efficacy (e.g., tamoxifen). The drug that satisfied our criteria was 17-dimethylaminoethylamino-17-demethoxygeldanamycin (17-DMAG) (Alvespimycin), a clinical-stage semisynthetic small-molecule derivative of the heat shock protein (Hsp90) inhibitor geldanamycin (25). Treatment of mice with established tumors with well-tolerated doses of 17-DMAG resulted in complete responses in most animals (Fig. 1*A* and *SI Appendix*, Fig. S1). Remission lasted between 6 and 20 wk, with an average of 11 wk of extension of mouse lifespan in the DMAG-treated mice (Fig. 1*A*), followed by tumor recurrence at the same site as the original tumor in 100% of animals. The antitumor effect of 17-DMAG was associated with inhibition of proliferation as determined by immunohistochemical (IHC) staining for Ki67 (Fig. 1*B*) and the induction of apoptosis manifested by IHC detection of activated caspase 3 (Fig. 1*C*). As expected for an Hsp90 inhibitor, the 17-DMAG treatment led to the activation of heat shock response (Fig. 1*D*) and a dramatic reduction in the levels of HER2/Neu protein (Fig. 1*E*), the stability of which is known to be dependent on Hsp90 (26). Thus, the MMTV-HER2/Neu mouse model appeared to be ideal for further assessment of the effects of NRTI on spontaneous breast tumor development, tumor response to chemotherapy using DMAG-17, and progression-free survival (time of refractory tumor relapses).

**Fig. 2.**
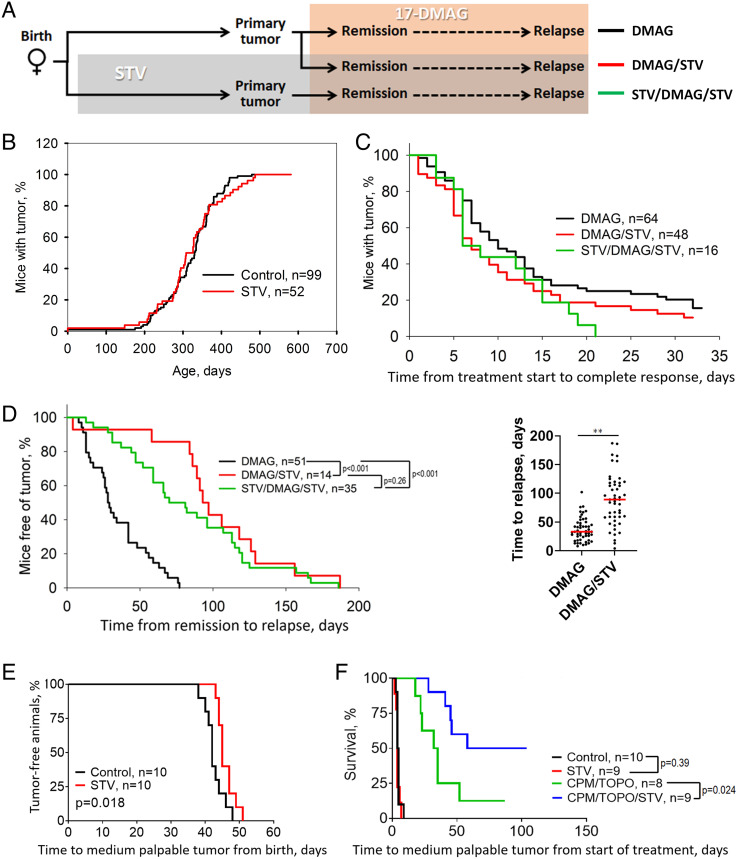
Effect of the NRTI STV on tumor development, the initial response to treatment, and relapse in MMTV-HER2/Neu and Th-MYCN mouse models. (*A*) Scheme of experiment with MMTV-HER2/Neu mice. Color codes on the right match the Kaplan–Meier curves in the panels *C* and *D*. (*B–D*) Effect of STV in drinking water and 17-DMAG IP injections on spontaneous breast tumor development in MMTV-HER2/Neu female mice. (*B*) Kinetics of tumor development in mice maintained on regular water or given STV-containing water from 8 wk of age. There were no statistically significant differences between the groups by Kaplan–Meier log-rank test. (*C*) Kinetics of tumor response to 17-DMAG treatment without STV treatment (“DMAG”) or with STV initiated at the time of tumor incidence (“DMAG/STV”) or 8 wk of age (“STV/DMAG/STV”). All mice had palpable tumors 5–100-mm^3^ size and started 17-DMAG treatment on day 0. There were no statistically significant differences among the groups by Kaplan–Meier log-rank test. (*D*) *Left* panel: Kinetics of 17-DMAG-resistant tumor relapse. All mice were free of palpable tumors on day 0 due to initial 17-DMAG treatment and continued 17-DMAG treatment for the duration of the experiment. STV-treated groups were started on STV-containing water at the time of DMAG treatment initiation (~44 wk; “DMAG/STV”) or 8 wk of age (“STV/DMAG/STV”). *Right* panel: dot plot of data from D with a red bar indicating median time to relapse. (*E*) Effect of STV in drinking water from weaning on spontaneous neuroblastoma development in Th-MYCN homozygous mice. (*F*) Effect of STV (in drinking water) on neuroblastoma growth and response to chemotherapy in Th-MYCN homozygous mice. (*P*-values for all survival curves were determined by Kaplan–Meier log-rank test).

To assess the effect of NRTI on tumor development, response to 17-DMAG and acquisition of treatment resistance, MMTV-HER2/Neu females were maintained on drinking water containing 1 mg/mL of STV (indicated as STV in figures). This dose was sufficiently efficacious to suppress the pathological effects of activated L1, as demonstrated in a recent work conducted in mice deficient for histone deacetylase Sirtuin 6 (*Sirt6*), a major contributor to the epigenetic repression of L1 (21). The experimental design is schematically illustrated in Fig. 2*A*. Mice at 2 mo of age were split into two groups that were then maintained either on regular water or water containing STV, and the time of tumor occurrence was registered. As shown in Fig. 2*B*, NRTI had no detectable effect on the time and incidence of breast tumor appearance and was well tolerated, as indicated by the lack of weight loss (*SI Appendix*, Fig. S1).

When the tumors become palpable (8–30 mm^3^), 17-DMAG treatment was initiated and continued until the end of the observation period (Fig. 2*A*). The STV-treated group continued with NRTI-containing water, while the control group was split into two subgroups, with either the presence or absence of STV in drinking water. Treatment with 17-DMAG had a strong antitumor effect resulting in complete response in ~90% of animals (Fig. 2*C*). Neither the speed of antitumor effect of 17-DMAG nor the proportion of complete responses was affected by STV regardless of whether STV treatment was started before or simultaneously with 17-DMAG.

Although NRTI was ineffective as a cancer prophylactic and had no direct antitumor activity in the MMTV-HER2/Neu model of breast cancer, it caused a dramatic effect on tumor relapse, significantly delaying tumor recurrence and prolonging progression-free survival, on average, from 25 d in the control group to 70 d in mice that received STV both prophylactically and during the time of 17-DMAG treatment, and to 95 d in mice that started drinking water with NRTI only from the time of commencement of the 17-DMAG treatment (Fig. 2*D*).

Histological assessment of tumors that had regrown and progressed despite continuous 17-DMAG treatment indicated visible domain structures: multiple tumor lobes separated by connective tissue, indicative of their oligoclonal nature. In contrast, late progressing tumors in STV-treated mice did not exhibit lobular domain structures, potentially reflecting a lesser number of tumor-initiating clones that survived 17-DMAG treatment (*SI Appendix*, Fig. S2).

We evaluated whether NRTI treatment caused any change in tumor immunoreactivity that might contribute to significant extension of relapse-free survival by enhancing either responses to 17-DMAG or immune surveillance post-17-DMAG. To do so, the overall amounts and proportions of various classes of immunocytes in the tumors were estimated by analyzing RNA sequencing data obtained for tumors from each category (untreated, STV-treated, and recurrent tumors that regrew following 17-DMAG and 17-DMAG + STV treatments). As evident from the results of this analysis (*SI Appendix*, Fig. S3), there were no significant differences in the amounts or composition of tumor-infiltrating immunocytes between the four tumor subsets, indicating that the observed extension of relapse-free survival reflects intrinsic properties of the tumor cell population as opposed to effects mediated via modulation of antitumor immune response.

To check whether the effect of NRTI on tumor recurrence following chemotherapy is a general phenomenon or is limited to the MMTV-HER2/Neu–17-DMAG model, we used another spontaneous cancer model with a well-characterized response to chemotherapy, the Th-MYCN mouse model (24, 27). In Th-MYCN mice, human *MYCN* expression is driven by a rat tyrosine hydroxylase (Th) promoter resulting in the development of neuroblastomas originating predominantly from abdominal ganglion structures. Tumors in these mice are highly sensitive to a combination of topotecan and cyclophosphamide (28), a regimen that is commonly used for the treatment of neuroblastoma patients and where many patients relapse following initial complete response. The results obtained from the use of STV in this model is shown in Fig. 2, panels *E* and *F*. NRTI in drinking water had a weak effect when administered in a prophylactic regimen, causing less than a 4-d-long delay in tumor development (from 42 to 46 d post-birth, on average) in Th-MYCN homozygous mice (Fig. 2*E*). STV treatment alone had no antitumor effect; however, if given simultaneously with chemotherapy, it dramatically delayed tumor relapses and increased the proportion of animals with no relapses within 100 d following cotreatment with topotecan and cyclophosphamide (Fig. 2*F*).

These observations suggest that NTRI belongs to a new category of potential oncology drugs that do not have direct anticancer activity but, if used in combination with anticancer treatment, can delay the acquisition of treatment resistance, thereby prolonging progression-free survival.

### L1 Expression Raises, and RT Inhibition Reduces, the Frequency of Drug-Resistant Variants in Cancer Cell Population.

The RT domain encoded by L1 is the main source of reverse transcriptase activity in mammalian cells. L1 RT not only drives retrotranspositions of L1 but also supports the expansion of SINEs and can generate cDNA copies of RNAs transcribed from pericentromeric satellite DNA and mRNAs (creating processed pseudogenes) (10). Expression of L1 is expected to be associated with genomic instability, a well-documented property of cancer cells and a likely mechanism underlying the extensive evolutionary plasticity and adaptability of tumors. Therefore, we hypothesized that the observed slowdown of tumor progression associated with NRTI treatment is attributed to inhibition of L1’s RT activity and mutagenic effects. To test this hypothesis, we focused our analysis on determining the frequency of drug-resistant variants appearing in a population of murine breast carcinoma following chemotherapy and evaluating the potential dependence on L1 activity. Since tumors from MMTV-HER2/Neu are poorly adaptable to tissue culture, we instead used the well-characterized murine breast carcinoma-derived cell line, 4T1 (29).

**Fig. 3.**
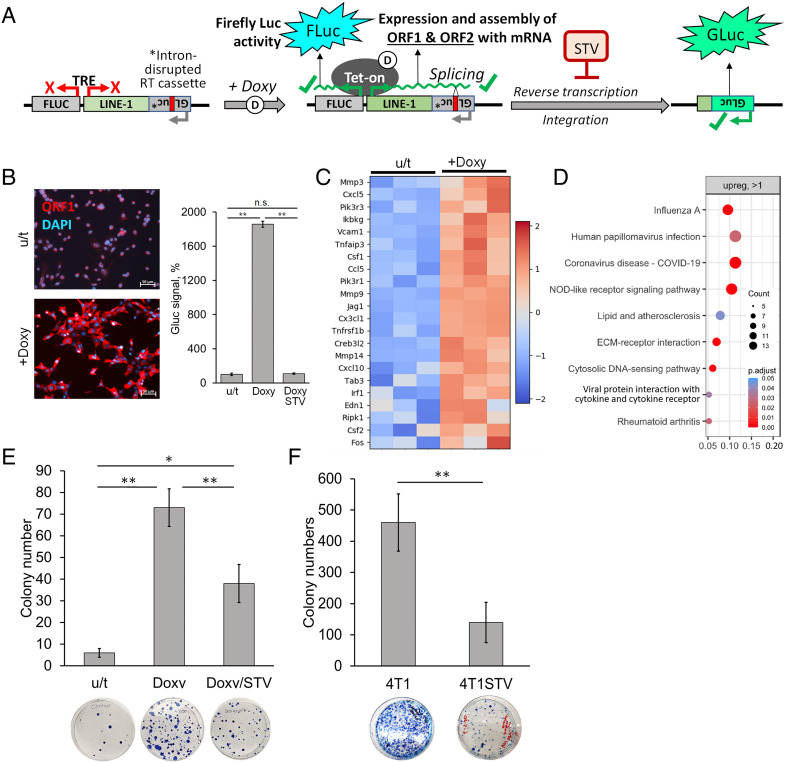
Phenotypic consequences of L1 induction in mouse 4T1 breast cancer cells. (*A*). Schematic description of the doxycycline-inducible L1 cassette stably integrated into 4T1-tetL1(GLucAI) cells. (*B*) Immunofluorescent staining shows induction of L1 ORF1 protein expression in 4T1-tetL1(GLucAI) cells following 48-h exposure to 400 ng/mL doxycycline (Doxy); *Right* panel shows the levels of Gaussia luciferase (Gluc) signal in the culture medium of 4T1-tetL1(GLucAI) cells following 48-h exposure to 400 ng/mL Doxy or 400 ng/mL Doxy and 10 μM STV. Untreated set at 100%. Error bars indicate SD in 3 of replicates, * means *P* < 0.05, ** means *P* < 0.001 (*C*) Heatmap of differentially expressed mRNAs identified by RNA-sequencing representing inflammatory response genes indicate activation of NF-κB and IFN type I pathways in 4T1-tetL1(GLucAI) cells following 48-h exposure to 400 ng/mL Doxy. The results of biological triplicates are shown as per-transcript z-score values for log2 of deseq2-normalized counts (based on RNA-seq data). (*D*) KEGG pathway enrichment for genes up-regulated with log2FC > 1 in the presence of Doxy (based on RNA-seq data). The results of biological triplicates are shown. (*E*) Colony formation assay with 4T1-tetL1(GLucAI) cells treated for 48 h with 1.5 μM 17-DMAG alone or together with 400 ng/mL Doxy or 400 ng/mL Doxy plus 10 μM STV. (*F*). Colony formation assay with 4T1 cells maintained in the absence or presence of 10 μM STV for 4 wk and treated with 1.5 μM 17-DMAG for 48 h. For *E* and *F*, Error bars indicate SD in 3 of replicates, * means *P* < 0.05, ** means *P* < 0.001. Photographs of representative plates for each treatment are shown below bar graphs.

To directly assess the effects of L1 expression on the frequency of drug-resistant cell variants, we generated a stable population of 4T1 cells with a tetracycline-inducible mouse L1 reporter construct that enables doxycycline-responsive, bidirectional expression of both firefly luciferase and a recoded mouse L1 construct harboring a retrotransposition readout cassette based on the secreted, superluminescent *Gaussia* luciferase (GLuc) (30). This L1 reporter line enables ultrasensitive detection of expressed retrotransposition events through the measurement of GLuc activity from the conditioned medium (Fig. 3*A*). The L1-encoding strand contains an intron at the 3′ end that disrupts a GLuc reading frame expressed on the opposite strand; thus, expression of functional GLuc protein requires the synthesis of an intact gene via the retrotransposition of the spliced, intronless L1 messenger RNA (mRNA). Inducibility of the L1 reporter is illustrated by immunofluorescent staining for L1 ORF1p in 4T1-tetL1(GLucAI) cells cultivated with or without doxycycline (Fig. 3*B*). The de novo synthesis and retrotransposition of intronless copies of L1(GLucAI) was proven by the induction of GLuc activity in the culture medium following doxycycline treatment. This effect is suppressed by nontoxic concentrations of STV added to the medium (Fig. 3*B*). As evident from global transcriptome analysis, the induction of L1 was associated with activation of NF-κB- and IFN-driven inflammatory pathways (Fig. 3 *C* and *D*). This response to L1 replication is expected, since products of reverse transcription are known to trigger inflammation via the cGAS-STING cytosolic DNA-sensing pathway (21).

We compared the frequency of cell variants capable of surviving 17-DMAG treatment in 4T1-tetL1(GLucAI) cells with and without L1 induction by doxycycline. To estimate the degree of drug resistance within a given cell population, we used clonogenic survival assays to calculate the proportion of cells capable of surviving a 48-h treatment of 1.5 μM 17-DMAG. In the absence of L1 induction, approximately 10^−4^ cells from the starting population retained clonogenicity following treatment. A nearly 10-fold increase in surviving clones was observed upon prior induction of the L1 cassette via doxycycline, an effect that was partially suppressed by STV (Fig. 3*E*). Neither doxycycline nor STV by themselves affected cell clonogenicity at the concentrations used (*SI Appendix*). These observations support a hypothesis that induction of L1 expression translates into an increased frequency of drug-resistant variants in a population of tumor cells.

Remarkably, long-term cultivation of 4T1 cells in the constant presence of STV results in a substantial (approximately, fourfold) reduction in the proportion of cells capable of clonogenic survival following 17-DMAG treatment, presumably due to suppression of background endogenous L1 activity (Fig. 3*F*).

### Mechanism of L1-Mediated Drug Resistance Is Associated with Activation of Proinflammatory Pathways.

To understand the mechanisms underlying L1-mediated drug resistance, we decided to characterize the fraction of 4T1 cells capable of surviving 17-DMAG treatment. The high frequency occurrence of such cells (~10^−4^) and unstable inheritance of their drug-resistant phenotype (*SI Appendix*, Fig. S3) argued against an acquired mutation(s)-based nature of resistance and suggested the involvement of epigenetic mechanisms underlying their properties. Therefore, we analyzed global transcriptomes of cell populations of drug-resistant variants enriched from several successive cycles of 17-DMAG treatment and selection. Besides the basal levels of mRNA expression, we also analyzed the transcriptional response of the original 4T1 cells and their 17-DMAG-selected variants to 17-DMAG treatment. Being an HSP90 inhibitor, 17-DMAG is expected to induce heat shock response, which could be manifested by activation of HSF1-driven transcriptional activation of genes encoding heat shock proteins. We were interested to see whether resistant cells would retain the ability to react to 17-DMAG. Finally, we planned to determine whether cells selected as 17-DMAG resistant would also possess resistance to other stresses.

**Fig. 4.**
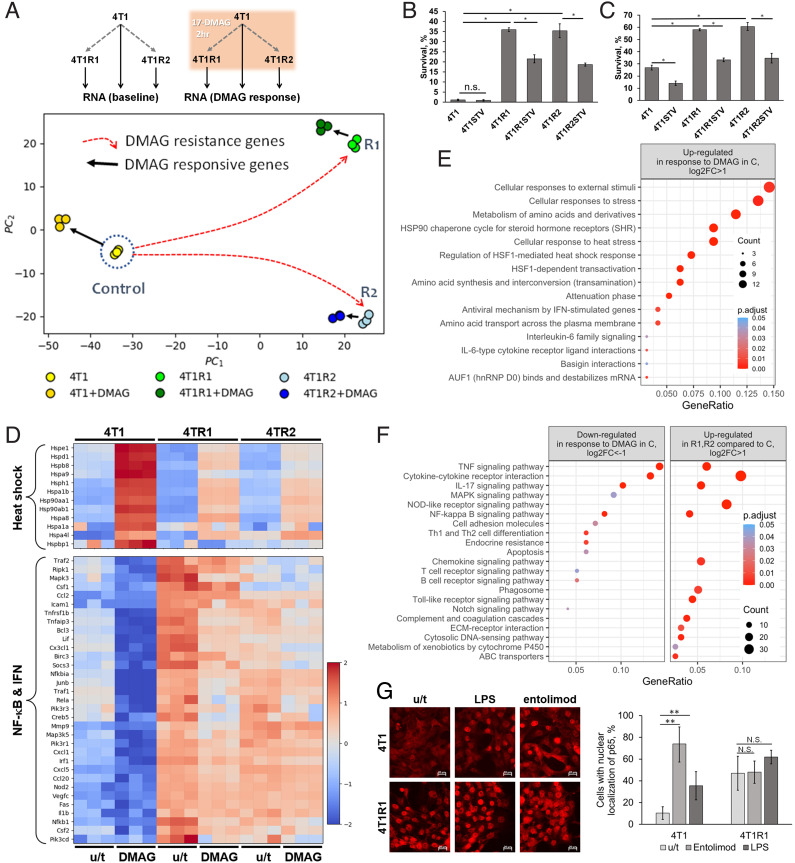
Analysis of transcriptome and phenotype of mouse 4T1 breast cancer cells selected for resistance to 17-DMAG. (*A*) Scheme of transcriptome analysis (*Top*) and PCA plot (*Bottom*). (*B* and *C*) Cytotoxicity assays were performed with parental 4T1 cells and two populations selected for 17-DMAG resistance (4T1R1 and 4T1R2). Cells were treated for 48 h with 1.5 μM 17-DMAG (*B*) or 250 ng/ml doxorubicin (*C*) in the absence or presence of 10 μM STV. For *B* and *C*, error bars indicate SD in 3 of replicates *, *P* < 0.05; n.s., not significant. (*D*) Heatmap derived from RNA-seq data indicating transcriptional changes associated with selection for 17-DMAG resistance and occurring in response to 17-DMAG treatment. *Top*: Hsp genes differentially expressed with adjusted *P*-value < 0.05, log2FC > 0.25; *Bottom*: genes from KEGG Pathway mmu04668 TNF signaling pathway differentially expressed with adjusted *P*-value < 0.05, log2FC > 0.25 C, control/parental 4T1 cells; R1 and R2, 17-DMAG-resistant selected populations 4T1R1 and 4T1R2. The results of biological triplicates are shown as per-transcript z-score values for log2 of deseq2-normalized counts. (*E*) KEGG pathway enrichment for genes up-regulated with log2FC >1 in response to 17-DMAG in 4T1 cells (based on RNA-seq data). (*F*) KEGG pathway enrichment for genes down-regulated with log2FC <−1 in 4T1 cells in response to 17-DMAG and up-regulated with log2FC >1 in 17-DMAG-resistant cells compared with parental 4T1 cells (based on RNA-seq data). (*G*) Immunofluorescent staining of NF-κB subunit RelA in 4T1 cells and their 17-DMAG-resistant variant 4T1R1 left untreated (control) or treated with NF-κB-activating agonists of TLR4 (31) or TLR5 (entolimod). The bar graph on the right shows quantitation of the immunofluorescence results in terms of the proportion of cells with nuclear RelA signal (indication of NF-κB activation). Mean ± SD for 10 view fields. **, *P* < 0.001; N.S., not significant.

The scheme used for the transcriptome analysis experiment is shown in Fig. 4*A*. First, several independent 17-DMAG-resistant cell populations were selected. Their resistance to the HSP90 inhibitor was confirmed, as shown in Fig. 4*B*, and treatment with STV caused partial but significant sensitization to the drug. Resistance of the selected population was not limited to 17-DMAG, as they displayed increased ability to survive treatment with doxorubicin, a drug with an unrelated mechanism of action. As with 17-DMAG, STV treatment caused a partial loss of resistance to doxorubicin (Fig. 4*C*). Notably, STV reduced the number of cells surviving doxorubicin treatment not only in 17-DMAG-resistant but also in parental 4T1 cells, suggesting that NTRI reduces the subpopulation of tumor cells that drive tumor recurrence following chemotherapy and thereby extends relapse-free survival. Thus, the effect of RT inhibition on tumor cell resistance to therapy is not limited to 17-DMAG and is likely to be true for other anticancer treatments. This conclusion is further supported from our above described results obtained in mouse neuroblastomas treated with topotecan and cyclophosphamide (Fig. 2*F*).

Total RNA sequencing data were analyzed using principal component analysis. Independently selected cell populations displayed similar changes in gene expression patterns as evident from their projection to the major Principal Component Analysis (PCA) axis (PC1) (Fig. 4*A*). Similar changes in gene expression patterns following 17-DMAG treatment were observed, indicating similar physiological responses to 17-DMAG inhibitor regardless of whether the cell population was resistant or not.

The analysis of differentially expressed genes defining the positions of cell populations on the PCA graph confirmed this interpretation and indicated the nature of the biological processes underlying drug resistance and 17-DMAG response mechanisms. Two major types of transcriptional responses were found to be involved in these processes. As expected from its mechanism of action (HSP90 inhibition), 17-DMAG treatment induced an HSP-1-driven heat shock response evident from the heat map of gene sets associated with differential gene expression revealed by PCA positions (Fig. 4*D*), their functional assessment revealed by KEGG analysis (Fig. 4*E* and *SI Appendix*, Fig. S4), and confirmed by qPCR (*SI Appendix*, Fig. S4). This observation indicates that the acquisition of 17-DMAG resistance was not associated with the loss of cell response and susceptibility to the HSP90 inhibitor.

Another major pattern of transcriptional response observed in all 17-DMAG-treated populations was a substantial reduction in the expression of genes encoding inflammation-associated factors commonly controlled by NF-κB (Fig. 4*D*). This is also an expected consequence of heat shock response known to be associated with general inhibition of nonchaperone gene translation (32) and known to suppress the NF-κB signaling and inflammation (33–35). Since constitutively active NF-κB is a universal property of cancer cells, essential for their viability (36), the reduction in NF-κB-driven transcription of prosurvival factors following 17-DMAG treatment is the likely reason for its anticancer effect.

While the direction of changes in transcriptional response to 17-DMAG (suppression of NF-κB-mediated transcription) was similar in both parental (17-DMAG-sensitive) and 17-DMAG-resistant cells (Fig. 4 *A* and *D* and *SI Appendix*, Fig. S5), the baseline levels of NF-κB transcription were dramatically different. As illustrated by Fig. 4 *D* and *F* and *SI Appendix*, Fig. S6, selection for 17-DMAG resistance was associated with a general increase in NF-κB signaling, indicative of the acquisition of a constitutive elevation of proinflammatory response by resistant cells. In fact, immunofluorescent staining demonstrated that the proportion of cells with constitutive presence of RelA (NF-κB subunit) protein in the nuclei of the resistant cell population was similar to that of sensitive cells following transient induction via treatment with the proinflammatory agents, TLR4 agonist lipopolysaccharide (31, 37) and TLR5 agonist, bacterial flagellin derivatibe entolimod (38) (Fig. 4*G*).

The remarkable coincidence of the functional profiles of genes up-regulated by 17-DMAG-resistant cells and genes that are down-regulated in response to 17-DMAG is indicated by comparison of KEGG analysis of differentially expressed genes (Fig. 4*F*). Thus, suppression of prosurvival NF-κB transcription in 17-DMAG-resistant cells in response to the drug, though similar in scale, was never as complete as in sensitive parental cells because of the much higher basal levels of NF-κB activity in the 17-DMAG-resistant cells, presumably enabling their survival during otherwise deadly stresses.

**Fig. 5.**
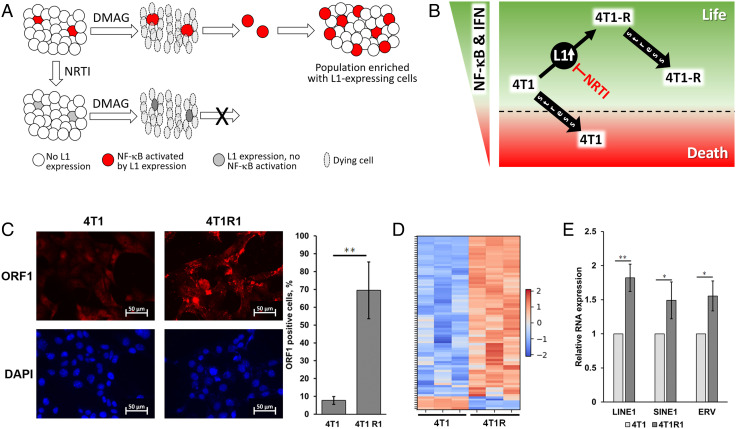
Drug resistance of 4T1 cells is associated with upregulation of retrotransposons. (*A*) Schematic description of the proposed mechanism by which drug-resistant cell populations arise due to induction of proinflammatory and prosurvival responses (e.g., NF-κB and IFN type 1 pathways) by activated L1. This mechanism can be blocked by NRTI-mediated inhibition of LI reverse transcriptase. 17-DMAG is shown as a representative anticancer drug. (*B*) Schematic illustration of the hypothetical mechanism of L1-mediated drug resistance is suggested by our results. Activation (desilencing) of L1 leads to increased basal levels of NF-κB and IFN type 1 pathways, which allows cells to survive stresses such as anticancer drug treatment. This prosurvival response can be abrogated by inhibition of L1 reverse transcriptase activity by NRTI. (*C*) Detection of L1 expression in parental 4T1 cells and in a cell population selected for 17-DMAG resistance (4T1R1) by immunofluorescent staining of L1 ORF1 protein. The bar graph on the right shows the quantitation of staining results. Mean ± SD for 10 view fields. **, *P* < 0.001. (*D*) Heatmap for transcripts of major classes of retrotransposons differentially expressed between 4T1 and 4T1R1 cells (based on RNA-seq data; adjusted *P*-value < 0.05). Results of biological triplicates are shown as per-transcript z-score values for log2 of deseq2-normalized counts. (*E*) RT-PCR-based quantitation of transcript levels of representatives of three major classes of retrotransposons in 4T1 and 4T1R1 cells. Transcript levels are shown normalized to the level in 4T1 cells (set at 1.0). Error bars indicate SD in four of replicates, * means *P* < 0.05, **, *P* < 0.001.

Similar effects (proinflammatory response and drug resistance) can be induced in 4T1 cells by activation of L1 in an inducible system (see Fig. 3 *C* and *D*), with the NRTI STV, in both cases, partially reverting the drug-resistant phenotype (Figs. 3*E* and 4 *B* and *C*). Furthermore, the cultivation of 4T1 cells in the presence of NRTI dramatically reduces the proportion of surviving cells following 17-DMAG treatment (Fig. 3*F*) and prolongs progression-free survival of tumor-bearing mice following initial treatment response (Fig. 2 *D* and *F*). These observations suggest (see the model in Fig. 5 *A* and *B*) that treatment with the HSP90 inhibitor favors survival of rare cells in the population with spontaneously active L1 transcription that was translated into a prosurvival inflammatory response. To test this assumption, we compared the expression of L1 in control and drug-resistant populations of 4T1 cells at the protein and RNA levels. As shown in Fig. 5*C*, immunofluorescent staining revealed L1’s ORF1 protein expression in the cytoplasm of the majority of drug-resistant cells and only in a small proportion of the parental population of 4T1 cells. Computational analysis of the abundance of transcripts revealed the elevated expression of various classes of retroelements, including L1, in resistant cell populations (Fig. 5*D* and *SI Appendix*, Fig. S7). These results were confirmed by quantitative RT-PCR (Fig. 5*E*).

## Discussion

Using in vitro and in vivo models, we found that the acquisition of treatment resistance by mouse breast cancer cells was associated with the selection of cells with the constitutively elevated activity of proinflammatory NF-κB and IFN type I pathways and elevated expression of retrotransposons, including L1. The interdependence of these properties is supported by i) the induction of an inflammatory response and acquisition of drug resistance following activation of L1 transcription in an inducible cell system, ii) the reversion of drug resistance by NRTI in vitro*,* and (39) a substantial extension of progression-free survival of tumor-bearing mice following initial response to therapy when therapy continues in the presence of NRTI. Thus, we conclude that selection for treatment resistance favors cells with increased inflammatory pathway activity due to spontaneous “retrobiome” activation.

Although most of the data were obtained by studying drug resistance following treatment with the HSP90 inhibitor 17-DMAG (chosen as the most convenient model of drug resistance acquired during continuous treatment), we believe that our conclusion that drug resistance is driven by the inflammatory response to an activated retrobiome reflects a general phenomenon because: i) the induction of L1 results in increased resistance not only to 17-DMAG but also to doxorubicin, with resistance reverted by NRTI treatment, ii) similarly, cells selected for resistance to 17-DMAG display resistance to doxorubicin, and (39) the extension of progression-free survival by NRTI was observed in another cancer model–spontaneous MYCN-driven neuroblastomas–that underwent treatment with a combination of drugs unrelated to HSP90 inhibition (cyclophosphamide and topotecan) and mimicking the clinical regimen. Thus, we are dealing with a drug resistance phenotype caused by the prosurvival function of inflammatory pathways induced by spontaneous desilencing of retrotransposons in rare cells in tumor cell populations (Fig. 5*B*). It is noteworthy that the described phenomenon does not assume that chemoresistance in vivo is associated with massive activation of L1 expression—it is sufficient that the chemotherapy provides a selective pressure that will favor the small proportion of cells with activation of the minor proportion of L1 copies that are functional and may arise stochastically through epigenetic dysregulation that exists as an inherent property of cancer. Provided that this subpopulation involves only a minor fraction of tumor cells and L1 activation can be reversible, such an increase could easily be below the detection threshold by transcriptome or proteome analysis of the bulk of the tumor.

The connection between retrobiome desilencing and inflammation has been demonstrated in numerous studies. First, we demonstrated activation of IFN by desilenced retrotransposon transcription following treatment of p53-null cells with a DNA demethylating agent, the phenomenon we named TRAIN (16). The association of malignant transformation with a proinflammatory pattern shift in the global transcriptome has been documented in the works from Benjamin Greenbaum’s group (40).

Mechanistically, stimulation of an inflammatory response to activated retrotransposons has been linked to the cGAS-STING pathway, an ancient, evolutionarily conserved mechanism of antiviral defense that in mammalian cells acts to recognize cytosolic DNA and induce IFN type I and NF-κB signaling (41). Activation of inflammatory signaling via cGAS-STING by desilenced L1 was demonstrated by John Sedivy’s lab in senescent cells (17) and in our collaborative work with Vera Gorbunova’s group, in tissues of mice deficient in *Sirt6* with impaired epigenetic repression of L1 elements (21). cGAS-STING-mediated response to L1 expression was claimed to be the main contributor to SASP (senescence-associated secretory phenotype) (42), considered as a major driver of aging-associated inflammation (21). In both studies, the ability of STV and lamivudine—NRTIs capable of inhibiting L1’s reverse transcriptase—to suppress inflammation was demonstrated, a finding that is consistent with a cGAS-STING-mediated mechanism and indicating the “druggability” of pathologies associated with L1 desilencing. Our present work adds to this list the acquisition of treatment resistance of tumors, the major property underlying cancer escape from therapy.

NRTI could only partially revert drug resistance induced by L1 activation. This partial effect of NRTI was also observed in another model of L1-driven pathology—in Sirt6-deficient mice: both STV and lamivudine delayed lethality and partially reduced inflammation (21) but were unable to normalize their phenotype. These observations suggest that the products of L1-mediated reverse transcription (synthesis of which is suppressed by NRTI) may not be the only mediators of prosurvival inflammatory response to L1 induction. Other mechanisms could be involved such as, for example, activation of cGAS-STING signaling by fragments of DNA generated by the activity of L1 ORF2 endonuclease (Fig. 5*A*). The principal ability of DNA damaging treatments to induce cGAS-STING has been reported (43). cGAS-STING-independent antiviral responses to retrobiome activation involving, for example, TLR3 or TLR9 (44) should be also considered.

The role of endogenous retrotransposons in cancer has been traditionally viewed in the context of their mutagenic activity, i.e., their ability to generate, via insertional mutagenesis, genetic variability in tumor cell populations, thus enabling tumor evolution. The main conclusion of the present work is that desilencing of retrotransposons can counteract anticancer treatment through activation of prosurvival inflammatory pathways and allowing tumors to regrow after lethal stresses, a mechanism that is distinct from retrotransposon-mediated mutagenesis. Furthermore, this mechanism is “druggable” since it can be partially reverted by NRTI and, prospectively, with other pharmacological (e.g., inhibitors of L1 endonuclease, STING, etc.) and immunotherapeutic (e.g., directed toward cells expressing L1 antigens) approaches targeting an activated retrobiome. In this regard, NRTIs capable of suppressing L1 reverse transcriptase represent a category of anticancer agents that do not have direct antitumor activity but are capable of suppressing tumor adaptability.

The suppression of tumor adaptability by inhibiting L1-mediated effects may not be limited to acquisition of treatment resistance but may involve other aspects of cancer progression (e.g., metastatic, angiogenic, metabolic properties), further contributing to the extension of progression-free survival. Recently results of Rajurkar et al. indicating prolonged (vs. historical control) survival of patients with advanced refractory colorectal cancer treated with NRTI lamivudine (45) strongly argues in favor of this assumption. This opportunity is currently being also explored in phase II clinical trial of NRTI lamivudine in patients with small-cell lung cancer currently ongoing at Roswell Park Comprehensive Cancer Center (ClinicalTrials.gov Identifier: NCT04696575). In summary, inhibitors of L1 are projected to have broad applications in oncology by slowing down tumor progression and improving the outcome of any anticancer treatment by extending progression-free survival following initial response.

## Materials and Methods

### Mice and Tumors.

FVB/N-TgN (MMTV-neu) 202Mul mice were obtained from (Jackson Laboratories, Bar Harbor, Maine). The colony is breeding in Roswell Park Comprehensive Cancer Center Animal Care facility. Eight-week-old female mice were used for experiments. Animal care was provided following institutional guidelines, then was reviewed and approved by the Institutional Animal Care and Use Committee. Animals were provided a commercial rodent diet (5% 7012 Teklad LM-485 Mouse/Rat Sterilizable Diet, Harlan) and sterile drinking water. Tumor incidence in MMTV-HER2/Neu mice was assessed via inspection of mammary pads for palpable masses. Mice were monitored every other day for new tumor formation. Masses were measured with calipers along the two perpendicular diameters. Tumor volume was calculated by: V(volume) = L(length) × W(width)^2^/2. Chemotherapy treatment started when the volume of the tumor was 8–30 mm^3^.

The Th-MYCN transgenic mouse model of neuroblastoma, involving targeted expression of the human *MYCN* transgene to neural crest cells by use of a tyrosine hydroxylase promoter has been previously described (Weiss et al. EMBO J, 1997). Homozygous Th-MYCN^+/+^ mice develop neuroblastoma within 7 wk from birth, with tumors that closely mimic the human form of the disease. All animal experimental procedures were approved by the University of New South Wales Animal Care and Ethics Committee according to the Animal Research Act, 1985 (New South Wales, Australia) and the Australian Code for the Care and Use of Animals for Scientific Purposes (2013). Th-MYCN mice were monitored for tumor growth by abdominal palpation, which is routinely performed in our laboratory by experienced personnel until one of the following occurred: development of a medium-sized abdominal tumor (~10 mm) or signs of a thoracic tumor including hind limb paralysis or labored breathing, or in the event of not relapsing with a tumor, until 20 wk of age.

### Treatment of Mice.

STV was provided in nonacidic drinking water to MMTV transgenic mice ad libitum starting at 2 mo of age or after prime tumors’ appearance. STV provided in nonacidified sterile water, pH 7, at 1 mg/mL results in a dose ~200 mg/kg/day, estimated by assuming consumption of 4 mL/mouse per day. As a control, mice were provided with nonacidified sterile water.

17-Dimethylaminoethylamino-17-demethoxygeldanamycin (17-DMAG) (20 mg/kg) dissolved in Dulbecco's phosphate-buffered saline (DPBS) buffer was administered to tumor-bearing mice as IP injections three times per week, treated in the presence or absence of STV in drinking water. The dose and injection frequency were selected as a slight modification of the treatment protocol described previously (46). Potential general toxicity of 17-DMAG alone or in combination with STV was determined by monitoring the body weight of mice every 2 wk throughout the course of treatment. Mice continued to receive 17-DMAG treatments until regrown tumors reached the end point size of 1,500 mm^3^, at which time mice were sacrificed.

For all Th-MYCN studies from weaning, homozygous Th-MYCN mice were randomized to STV or nonacidified sterile water at 3 wk of age. Mice were monitored for tumor growth by abdominal palpation, which is routinely performed in our laboratory by experienced personnel until one of the following occurred: development of a medium-sized abdominal tumor (~10 mm) or signs of a thoracic tumor including hind limb paralysis or labored breathing, or in the event of not relapsing with a tumor, until 20 wk of age. For studies with chemotherapy, homozygous Th-MYCN mice were randomized into groups upon the development of a small palpable tumor (~5 mm), at ~5–6 wk of age. Chemotherapeutic agents were delivered for 5 d, intraperitoneally at 10 mg/kg/day for cyclophosphamide and 0.5 mg/kg/day for topotecan, with or without STV, as described above. Mice were monitored until one of the described end points.

Termination of MMTV and Th-MYCN mice, for animals reaching one of the above end points, was performed by CO_2_ overdose. Tumors from mice of different treated groups were excised and half of the tumor fixed in 10% formalin and the other half frozen (−80°C).

**Reagents** are listed in *SI Appendix*, Table S1.

### Cell Culture.

The 4T1 mammary carcinoma tumor cell line was obtained from the American Type Culture Collection. 4T1 cells were maintained in Dulbecco's Modified Eagle Medium (DMEM)/F12 medium (Corning) containing 50 mL/L fetal bovine serum (Gibco), 100,000 U/L penicillin, and 100 mg/L streptomycin (Pen Strep) (Gibco) at 37°C in a humidified atmosphere with 5% CO_2_. 4T1 cells resistant to 17-DMAG were generated by passing 4T1 cells through three rounds of exposure to 1.5 μM 17-DMAG for 48 h and two populations (4T1R1 and 4T1R2) were used for further experiments. Staining with methylene blue was used to evaluate the cytotoxicity of 17-DMAG in 4T1 cells and confirm the development of drug resistance in 4T1R cells at different time points during the process. The culture conditions for 4T1R cells were the same as those of the parent cell line.

To generate a stable population of 4T1 cells carrying an integrated tetL1-GLucAI construct named 4T1-tetL1(GLucAI) cells, 4T1 cells were cotransfected with the tetL1-GLucAI donor plasmid (pBH201, described in detail below) and the Super PiggyBac Transposase Expression Vector (System Biosciences) at a 5:1 plasmid ratio, as recommended by the manufacturer’s protocol, to facilitate PiggyBac transposase-mediated integration of the ITR-flanked donor cassette. Transfections were performed using GenJet Plus reagent (SignaGen) with a total of 3-μg plasmid DNA per 10-cm dish, following the manufacturer’s protocol. Seventy-two hours after transfection, cells were selected for 3 d in 5 μg/mL Blasticidin S (Thermo). After two passages, an additional 72-h selection was performed to ensure the acquisition of a population with stable integration of the tetL1-GLucAI cassette. The culture conditions for 4T1-tetL1(GLucAI) cells were the same as those of the parent cell line.

### Recombinant L1-Expressing Inducible Cassette.

The inducible mouse L1 reporter plasmid used to generate 4T1 tetL1(GLucAI) cells (pBH201) contains elements originating from the XLone-GFP plasmid (Addgene #96930; a gift from Xiaojun Lian) (47). XLone-GFP harbors an "all-in-one" third-generation tetracycline-inducible system flanked by PiggyBac transposase-specific short inverted terminal repeats (ITRs) that enables integration of the construct into chromosomal sites containing the target sequence "TTAA" via a cut-and-paste mechanism (47). The inducible system flanked by the ITRs consists of two promoters in opposite orientations: an EF1α promoter driving constitutive expression of the Tet-On® 3G transactivator and blasticidin resistance genes, and a TRE3GS promoter that drives doxycycline-responsive expression of enhanced green fluorescent protein (EGFP). Through a series of successive PCR-based cloning steps performed using Genscript, the following modifications were made to XLone-GFP in order to generate pBH201: i) EGFP and associated SV40 polyA sequence were replaced with a recoded mouse L1 sequence (ORFeus) (48) synthesized by GenScript, encoding both ORF1 and ORF2; ii) a new GLuc-based retrotransposition readout cassette (GLucAI) synthesized by GenScript, was inserted within the 3′-UTR of L1; GLucAI is encoded in the opposite orientation of L1 and consists of SV40 enhancer and early promoter-driven expression of a superluminescent variant of *Gaussia princeps* luciferase (F89W/I90L) (30) with a C-terminal hemagglutinin (HA) tag (49), a disruption of the GLuc gene at nucleotide 117 by an antisense-oriented 133-bp chimeric intron (human β-globin donor and immunoglobulin heavy chain acceptor), and a herpes simplex virus thymidine kinase poly(A) signal; iii) in addition, the synthesized fragment contained an SV40 polyA signal in the sense orientation of L1 downstream of the GLucAI cassette for termination of the L1-GLucAI transcript, and iv) the TRE3GS promoter was replaced with a fragment cloned from the pTRE3G-BI-Luc Control plasmid (Takara Bio) containing a bidirectional, tetracycline-inducible promoter (P_TRE3G-BI_) and firefly luciferase gene with downstream SV40 polyA sequence.

### L1 Retrotransposition Assay.

To quantitatively assess retrotransposition activity in cells, 4T1-tetL1(GLucAI) were plated at 10^6^ per 10-cm plate, and the expression of the integrated tetL1-GLucAI cassette was induced by the addition of doxycycline (Doxy) to a final concentration of 400 ng/mL in the culture medium. After 48 h, cells were collected for RNA isolation (*SI Appendix*, *Materials and Methods*), and GLuc activity in the conditioned medium (indicative of retrotransposition) was measured using a microplate luminometer immediately following the addition of an equal volume of 2x GLuc reagent (50 μM coelenterazine (GoldBio) in D-PBS containing 300 mM sodium ascorbate (Sigma) and 0.2% Triton X-100 (Sigma)).

### Global Transcriptome Analysis.

The quality of the RNA sequencing data was assessed via FastQC (http://www.bioinformatics.babraham.ac.uk/projects/fastqc). Reads were aligned to the mouse reference genome (University of California, Santa Cruz (UCSC) mm10/GRCm38) with STAR RNA-seq aligner (50) using annotation from the same source. Reads were counted using featureCounts (51) using the same annotation. Normalized counts and differential expression (DE) of genes were obtained using DESeq2 (52). All sequencing data sets were deposited in NCBIs Gene Expression Omnibus (GEO, http://www.ncbi.nlm.nih.gov/geo/) and are available as a SuperSeries under accession GSE217043.

Further counts and DE comparison, analysis, and visualization were performed using both Python and R programming languages. Normalized counts were used for data visualization using PCA, which is a commonly used dimensionality reduction technique allowing to visualize the main variation in the data (53). Counts were also used for plotting heatmaps, with each row representing a gene and each column representing a sample; in all cases, the coloring was based on per-gene z-score values for log2 of normalized counts.

Kyoto Encyclopedia of Genes and Genomes (KEGG) enrichment was performed with R clusterProfiler package software (54) and presented as dot plots, with the dot size representing gene count enriched in the pathway, and the dot color showing the enrichment significance.

To quantify transposable element expression from alignment results, TEtranscripts (55) was used to measure the abundance of different transposable elements by combining transcripts of all instances of the same TE. After obtaining TE counts from TEtranscripts, once again deseq2 was applied for getting normalized counts and DE information.

### Statistical Analysis.

Survival curves were generated using Kaplan–Meier estimators and compared using the log-rank test in SigmaPlot 11. Data for cell survival, GLuc signal, qRT-PCR, p65 protein localization were analyzed by unpaired Student's two-tailed *t* test and are expressed as mean ± SD. *P* values ≤0.05 were considered statistically significant. All statistical analyses were performed using GraphPad Prism 9 software.

## Supplementary Material

Appendix 01 (PDF)

## Data Availability

The quality of the RNA sequencing data was assessed via FastQC (https://www.bioinformatics.babraham.ac.uk/projects/fastqc). Reads were aligned to the mouse reference genome (UCSC mm10/GRCm38) with STAR RNA-seq aligner using annotation from the same source. Reads were counted using featureCounts using the same annotation. Normalized counts and differential expression (DE) of genes were obtained using DESeq2. Data have been deposited in NCBI’s Gene Expression Omnibus (GEO, https://www.ncbi.nlm.nih.gov/geo/) (GSE217043). Vectors and genetically modified cells can be provided upon request under Material Transfer Agreement.
