# Supplementary material for: Inflammatory response to retrotransposons drives tumor drug resistance that can be prevented by reverse transcriptase inhibitors
Source: Proc Natl Acad Sci U S A. 2022 Nov 30;119(49):e2213146119. doi: 10.1073/pnas.2213146119 (PMC9894111; doi:10.1073/pnas.2213146119)
Supplement: Supplementary file 1 — Appendix 01 (PDF) [file pnas.2213146119.sapp.pdf]

Induction of a pro-survival inflammatory response to retrotransposons drives tumor resistance to treatment that can be prevented by reverse transcriptase inhibitors

Ksenia A. Novototskaya-Vlasova, Nickolay S. Neznanov, Ivan Molodtsov, Brandon M. Hall, Mairead Commane, Anatoli S. Gleiberman, Jayne Murray, Michelle Haber, Murray D. Norris, Katerina I. Leonova, and Andrei V. Gudkov

Supplementary Materials

Content	Pages
Supplementary Figures S1 – S8	**2 – 7**
Supplementary Materials and Methods	**8 – 9**
Supplementary Tables S1 and S2	**10 – 12**

Supplementary Figures

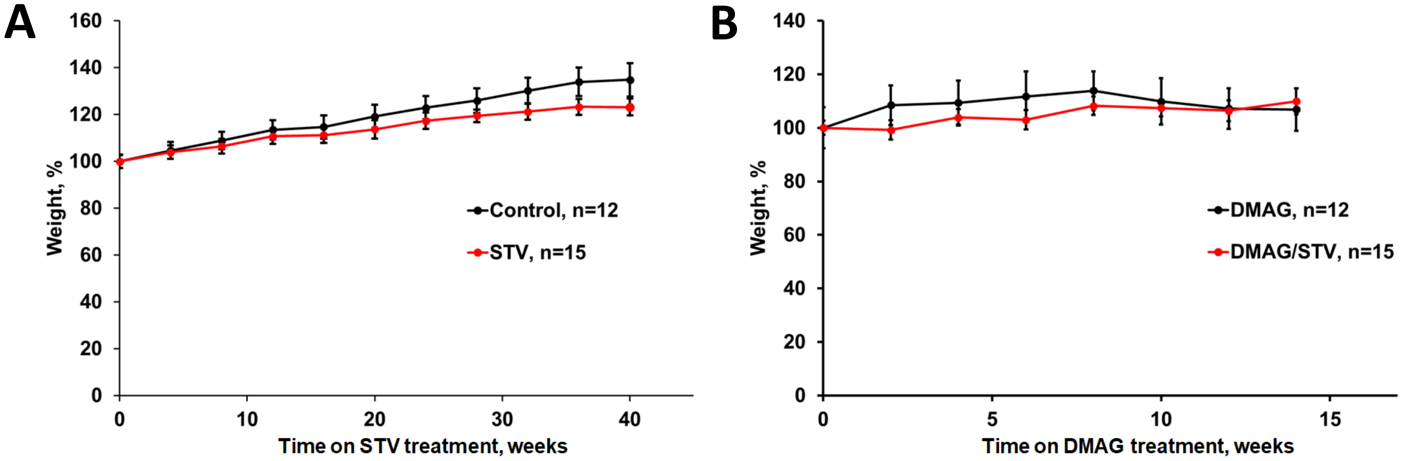

Supplementary Fig. S1. Effects of stavudine (STV) (**A**), 17-DMAG (DMAG) treatment, and combination of both (DMAG/STV) (**B**) on mouse weight dynamic. Treatment schedules and doses are described in Materials and Methods. There are no statistically significant differences between groups in either panel. The weights of the same animals are shown in panels A and B. All mice developed tumors at times later than 40 days on STV (i.e., panel A shows the weight dynamic of tumor-free mice). 17-DMAG treatment was initiated following tumor development (panel B).

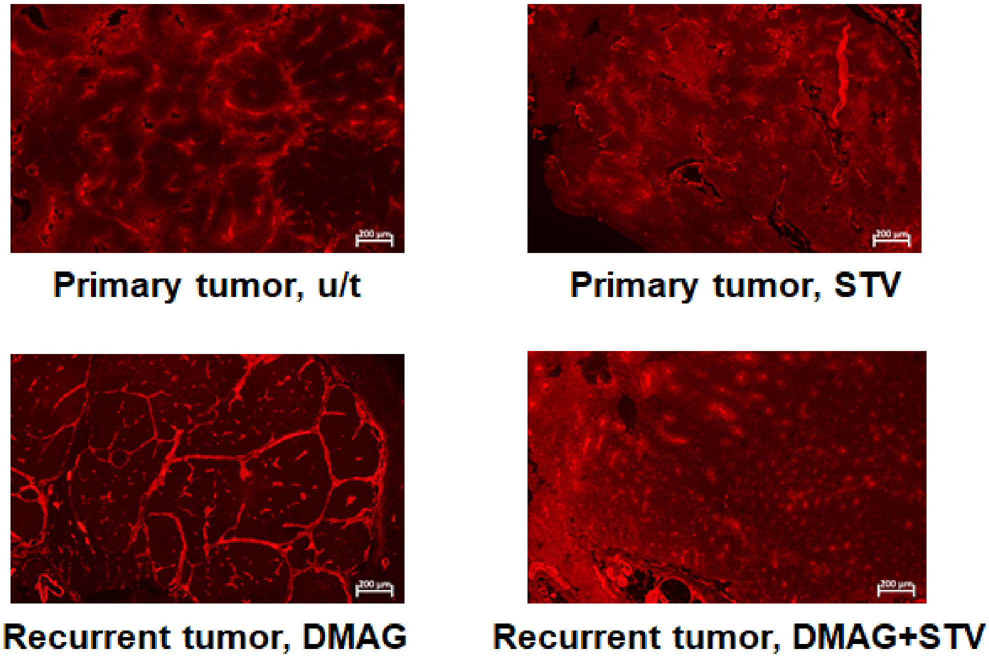

Supplementary Figure S2. Representative images of histological structure of primary tumor developed in untreated (u/t) and in stavudine-treated (STV) mice and recurrent tumors regrown following complete response to 17-DMAG treatment (DMAG), in the presence and in the absence of stavudine (STV) in drinking water. Immunofluorescent staining for smooth muscle actin (SMA) indicates the lobular structure of the tumor regrown following 17-DMAG treatment alone consistent with its polyclonal origin.

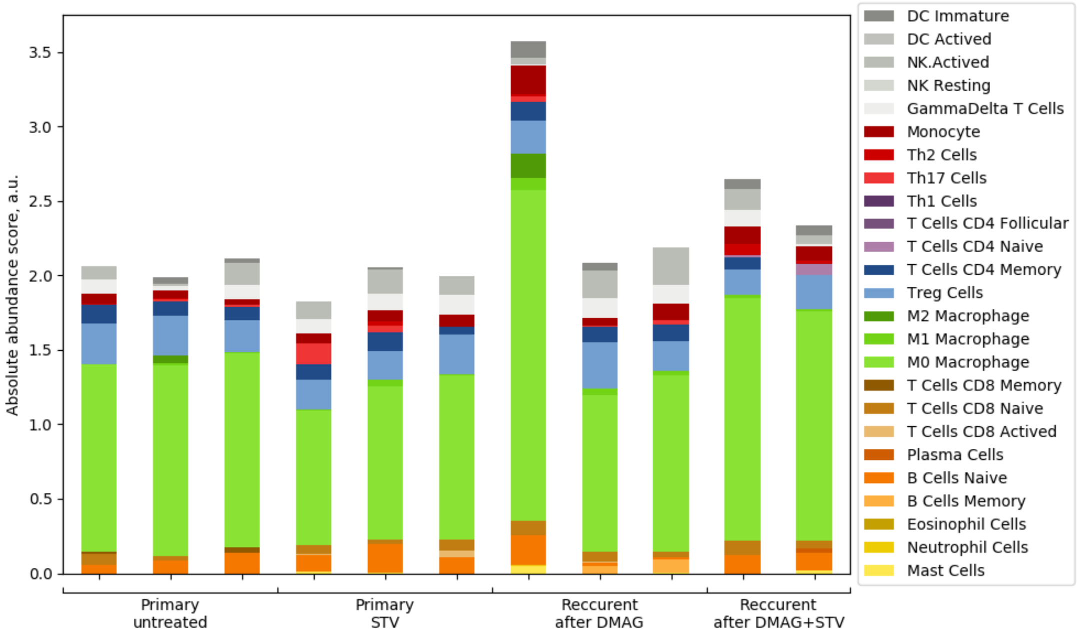

Supplementary Figure S3. Tumor infiltrating lymphocytes abundances estimated from RNA sequencing. Absolute scores from CIBERSORTx for 25 mouse immune cell types are provided for samples from 4 different tumor types: primary tumor developed in untreated ('primary untreated') and in stavudine-treated ('primary STV') mice and recurrent tumors regrown following complete response to 17-DMAG treatment, in the presence and in the absence of stavudine in drinking water ('reccurent after DMAG' and 'Reccurent after DMAG+STV', correspondingly). For inference of immune cell composition of samples, we used CIBERSORTx online analysis platform (https://cibersortx.stanford.edu/) (1) was used to analyze gene expression profiles. Mouse-specific leukocyte signature matrix from ImmuCC (2) developed to infer the proportions of 25 mouse immune cell types was used with the CIBERSORTx framework. After running CIBERSORTx, we obtained the abundance (absolute values in arbitrary units) of 25 subsets of immune cells in each sample with a p-value measuring the confidence of the results for the deconvolution being <0.05 in all samples. The resulting immune cell subpopulation frequencies estimated by CIBERSORTx demonstrated no significant differences between different tumor types.

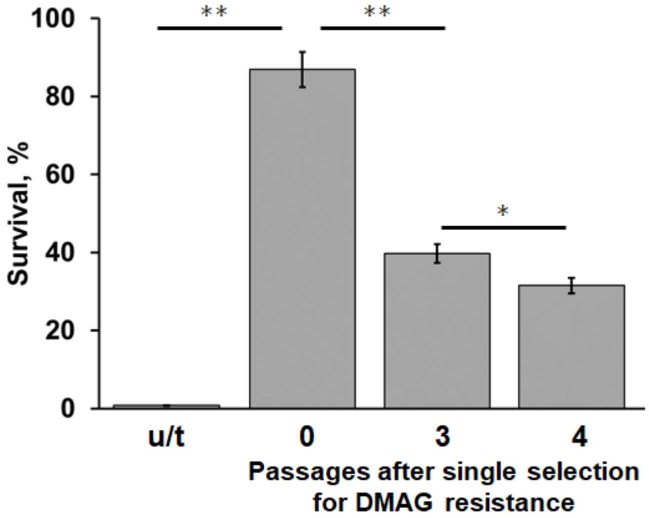

Supplementary Fig. S4. Gradual decline in the proportion of 17-DMAG-resistant cells during propagation of 4T1 cell population in the absence of Hsp90 inhibitor following single selection. Relative cell numbers (Survival, %) compared to 17-DMAG-untreated cells were determined using methylene blue assay. The conditions of 17-DMAG selection are described in Materials and Methods.

A

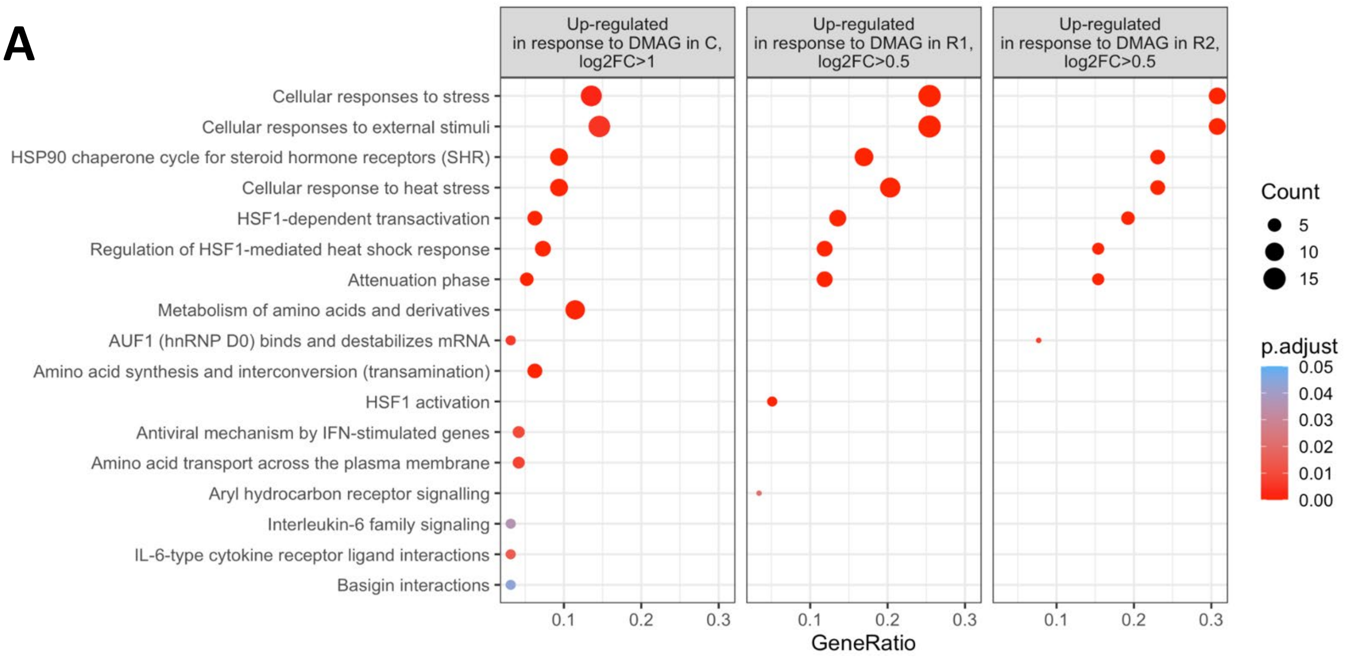

B

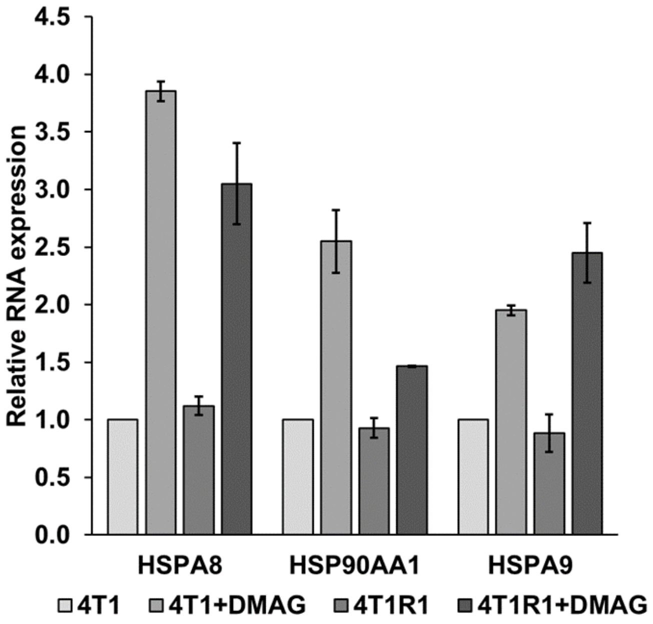

Supplementary Fig. S5. **A.** KEGG pathway enrichment for genes upregulated with log2FC>1 in response to 17-DMAG in 4T1 cells and with log2FC>0.5 in 4T1R1 and R2 resistant cells (based on RNA-seq data). **B.** quantitative RT-PCR-based validation of gene expression differences in 4T1 and 4T1R1 cells without or with 1h 17-DMAG treatment for the indicated heat shock protein genes. Transcript levels are shown normalized to the level in untreated 4T1 cells (set at 1.0). Error bars indicate standard deviation in 4 of replicates.

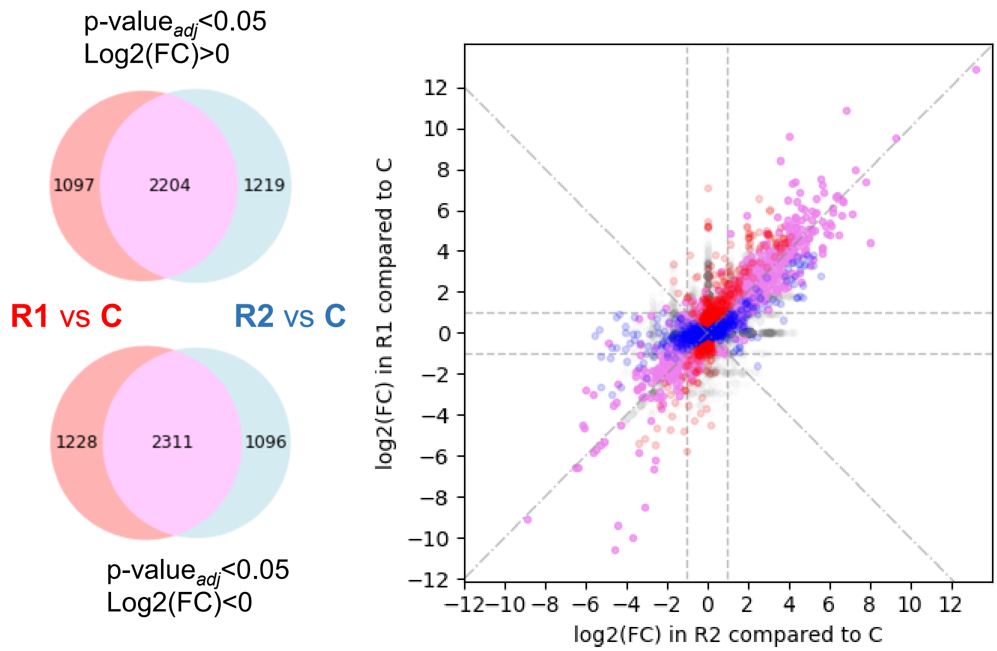

Supplementary Fig. S6. Venn diagrams for the genes, which are up-regulated (p-valueadj<0.05, log2(FC)>0; top) or down-regulated (p-valueadj<0.05, log2(FC)<0; bottom) in 4TR1 (red) and 4TR2 (blue) cells vs parental 4T1 and violet used for intersection. Scatter plot for per-gene log2(FC) in 4TR1 and 4TR2. Red dots show genes which are DE with pvalueadj<0.05 in 4TR1 only, blue – in 4TR2 only, and violet – genes which are DE with p-valueadj<0.05 both in 4TR1 and 4TR2. Dashed lines show log2(FC) = -1/1 thresholds; dash-and-dotted lines show thresholds of equal absolute log2(FC) values

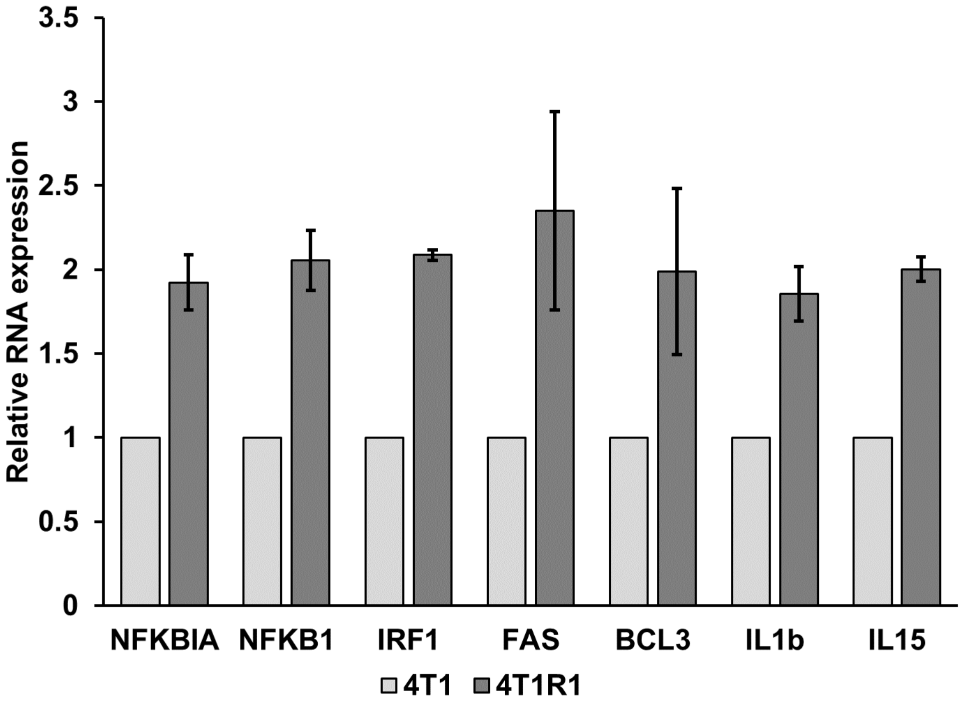

Supplementary Fig. S7. Quantitative RT-PCR-based validation of gene expression differences in 4T1 and 4T1R1 cells for the indicated NF-κB pathway related genes. Transcript levels are shown normalized to the level in untreated 4T1 cells (set at 1.0). Error bars indicate standard deviation in 4 of replicates.

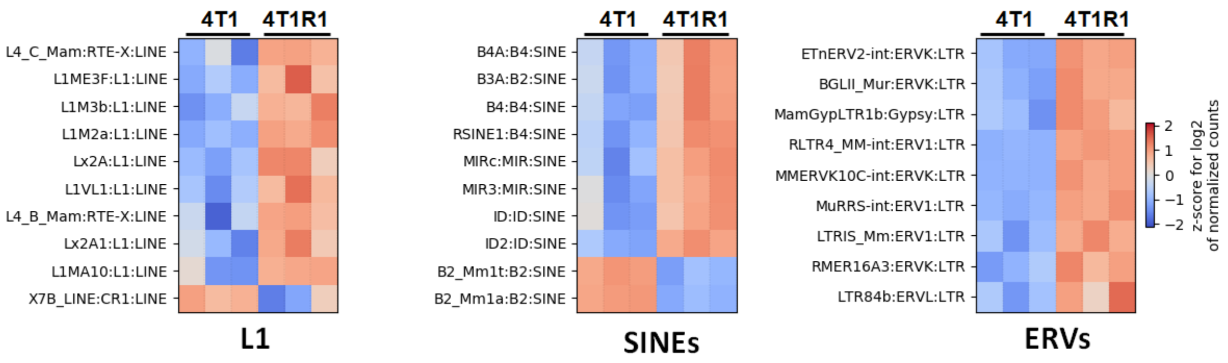

Supplementary Fig. S8. Heatmap for transcripts of major indicated classes of retrotransposons differentially expressed between 4T1 and 4T1R1 cells (based on RNA-seq data; adjusted p-value < 0.05). Results of biological triplicates are shown as per-transcript z-score values for log2 of deseq2-normalized counts.

References

1. 4. C. B. Steen, C. L. Liu, A. A. Alizadeh, A. M. Newman, "Profiling cell type abundance and expression in bulk tissues with CIBERSORTx" in Stem Cell Transcriptional Networks. (Springer, 2020), pp. 135-157.
2. Z. Chen *et al.*, Inference of immune cell composition on the expression profiles of mouse tissue. *Scientific reports* **7**, 1-11 (2017).

Supplementary Materials and Methods

Cytotoxicity Studies

Cells were plated at a density of 10,000 cells/well in 6-well plates and the treatment agents (1.5µM 17-DMAG, 250 ng/ml doxorubicin (Sigma) or combination of 1.5µM 17-DMAG and 10 µM STV or 250 ng/ml doxorubicin and 10 µM STV) were added. After 48h of treatment, cells were fixed using 1 mL per well staining solution (50% methanol, 50% water, 1% methylene blue dye). After incubation for 1h at room temperature, plates were rinsed three times with water and air-dried. 1% sodium dodecyl sulfate (SDS) solution was then added to the wells to solubilize the protein-bound dye and the optical density was measured using a plate reader (Perkin Elmer Victor 3x) in 96-well format.

Colony formation Assay

4T1 cells were passaged in the presence of 10 µM STV in parallel with untreated cells for four weeks. After this period colony formation assay was performed. Cells were seeded at a density of 10^6 cells per 10-cm Petri dish in triplicate. On the next day, cells were treated with 1.5 µM 17-DMAG. After 48h of 17-DMAG treatment, the medium was replaced with a fresh medium and the cells were cultured for another 5 days. On day seven, cells were fixed and stained with 1% methylene blue solution after washing with PBS. The number of colonies was counted. 4T1 tetL1(GLucAI) were passaged in three variants: untreated, with 400 ng/ml doxycycline (Doxy) (Sigma), and with combination of 400 ng/ml Doxy and 10 µM STV for four weeks. The concentrations of Doxy and STV used did not affect cell clonogenicity. The same experiment for DMAG-resistant cell variants was performed.

Quantitative RT-PCR

4T1 and 4T1R1 cells were seeded at a density of 10^6 cells per 10 cm Petri dish in duplicate. On the next day, half of the cells were treated with 1.5 µM 17-DMAG. After two hours of 17-DMAG treatment, cells were collected and total RNA was isolated using Trizol(Thermofisher Scientific, Fremont, CA) and then treated with DNase I (Sigma) following the manufacturer's protocol. Quantitative RT-PCR (qRT-PCR) was performed on a CFX384 Touch Real-Time PCR Detection System using the iTaq Universal One-Step Kit (BioRad) and 5 ng of RNA per reaction. Four replicate reactions were performed for each sample. GAPDH was used as a housekeeping gene for data normalization. Primer sequences are provided in Supplementary Table 2.

Western immunoblotting

Tumor and normal mammary tissues were sonicated in ice-cold RIPA buffer (Sigma) plus protease inhibitors (PIC) (Sigma) and cleared by centrifugation in a Beckman tabletop (20min, 12,000 rpm, 4°C). The protein concentrations of lysates were determined with the Quick start Bradford reagent (Bio-Rad), using bovine serum albumin (BSA) (Sigma) as a standard. For Western blots, 20 µg of total tissue protein were loaded per well. Precision plus dual color protein molecular weight markers (Bio-Rad) were also loaded. Proteins were then transferred to Immun-Blot PVDF membranes (Bio-Rad). After blocking in 2.5% non-fat dry milk (RPI) TBST (10 mM Tris-HCl (ThermoFisher) pH 7.5, 150 mM NaCl (ThermoFisher), 0.1% Tween-20 (Sigma)) for 1h at room temperature, blots were incubated with primary antibodies against mouse HER2 (1:1000 dilution, Santa Cruz) or HSP70/72 (1:1000 dilution, Enzo) followed by horseradish peroxidase (HRP)-conjugated secondary anti-rabbit antibodies (1:1000 dilution, Santa Cruz). Alternatively, HRP-conjugated primary antibodies against β-actin (1:10,000 dilution, Sigma) were used for probing protein loading controls. Membranes were washed three times with TBST, then developed by ECL (Perkin-Elmer) and documented using the ChemiDoc MP Imaging System (BioRad).

Immunostaining of cells and tumor sections

Cells were fixed for 15 min in 4% paraformaldehyde (PolySciences) in PBS (Corning) at 4°C, washed 3x in PBS, and incubated with blocking solution (5% normal donkey serum (Sigma), 0.2% triton x-100 (Sigma), PBS(Corning), 1% glycine (Sigma)) for 15 min. After fixation, cells were stained with primary antibodies against LINE-1 ORF1 (1:400 dilution, Millipore) or NF-κB p65 (1:200 dilution, Cell Signaling). Cells were then washed with 5 changes of PBS for 6–8h total at room temperature and stained overnight with secondary donkey anti-mouse antibody against whole molecule IgG conjugated with Cy3 (1:1000 dilution, Sigma) or AlexaFluor488 (1:1000 dilution, Thermofisher). Nuclei were counterstained with DAPI (Thermofisher). Samples were washed with several changes of PBS for a total of 4h, cleared, and

mounted with ProLong Gold antifade reagent (Thermofisher). Images were collected with a Zeiss AxioImager 2 fluorescent microscope equipped with an AxioCam 702 digital camera using ZEN software 2.6 version.

Tumors collected from in vivo study were dehydrated, embedded in paraffin, and sectioned into 5–10-micron sections using a microtome. The sections were gently placed on positively charged slides, deparaffinized, and rehydrated using xylene, ethanol, and double-distilled water. The sections were then boiled for 30 min in R-Universal antigen retrieval buffer (Electron Microscopy Sciences) for antigen unmasking and incubated in 3% hydrogen peroxide solution for 15 min. After blocking with 5% donkey serum (Sigma), sections were incubated overnight with primary antibodies for HER2 (1:200 dilution, DAKO), HSP70 (1:100 dilution, Cell Signaling), Ki67 (1:200 dilution, Thermo Scientific), Caspase 3 (Asp175) (1:100 dilution, Cell Signaling), Survivin (1:100 dilution, Cell Signaling). On the next day, the slides were washed and stained using the Ultravision ONE HRP polymer kit (Thermofisher) per the manufacturer's instructions. The sections were then counterstained with Mayer's hematoxylin (Sigma), dehydrated, mounted using Fisher Chemical Permount Mounting Medium (Thermofisher), and imaged using a Zeiss AxioImager 2 microscope equipped with an AxioCam 512 digital camera and ZEN software 2.6 version. Immunofluorescent staining for SMA was performed as described above except that after blocking with 5% donkey serum (Sigma), slides were incubated with a Cy3-conjugated mouse monoclonal antibody against SMA (1:1000 dilution, Sigma) for 30 min, washed and imaged.

RNA isolation

10-50 mg of frozen tissue samples, with the addition of 700 µl of Qiazol reagent, are first homogenized using Navy Rhino tubes in a Bullet Blender Homogenizer (Next Advance) for five minutes. The homogenate is then removed and incubated in a new tube at room temperature. After addition of chloroform, the homogenate is then separated into aqueous and organic phases by centrifugation. RNA partitions to the upper, aqueous phase, while DNA partitions to the interphase and proteins to the lower, organic phase or the interphase. The upper, aqueous phase is extracted, and ethanol is added to provide appropriate binding conditions for all RNA molecules from 18 nucleotides upwards. The sample is then applied to the miRNeasy Mini spin column, where the total RNA binds to the membrane and phenol and other contaminants are efficiently washed away. On-column DNAse digestion is also performed to remove any residual genomic DNA contamination followed by additional washes. High quality RNA is then eluted in 60 µl of RNase-free water.

Quantitative assessment of the purified total RNA is then accomplished by using a Qubit Broad Range RNA kit (Thermofisher), and concentration is determined by Ribogreen fluorescent binding to isolated RNA. The RNA is further evaluated qualitatively using RNA Nanotape on the 4200 Tapestation (Agilent technologies), where sizing of the RNA is determined, and a qualitative numerical score (RINe) is assigned.All RNA sequencing experiments were done in triplicates.

RNA-sequencing (RNA-seq)

Sequencing libraries were prepared with the RNA HyperPrep Kit with RiboErase (HMR) kit (Roche Sequencing Solutions) from 500ng total RNA. Following the manufacturer's instructions, the first step depletes rRNA from total RNA. The remaining RNA is DNase-digested to remove any genomic DNA contamination. Samples are then purified, fragmented, and primed for cDNA synthesis. Fragmented RNA is then reverse transcribed into first strand cDNA using random primers. The next step removes the RNA template and synthesizes a replacement strand, incorporating dUTP in place of dTTP to generate ds cDNA. Pure Beads (KAPA Biosystems) are used to separate the ds cDNA from the second strand reaction mix resulting in blunt-ended cDNA. A single 'A' nucleotide is then added to the 3' ends of the blunt fragments. Multiple indexing adapters, containing a single 'T' nucleotide on the 3' end of the adapter, are ligated to the ends of the double-stranded cDNA, preparing them for hybridization onto a flow cell. Adapter-ligated libraries are amplified by PCR, purified using Pure Beads, and validated for appropriate size on a 4200 TapeStation using D1000 Screentape (Agilent Technologies, Inc.). The DNA libraries are quantitated using the KAPA Biosystems qPCR kit (Sigma) and are pooled together in an equimolar fashion, following experimental design criteria. NovaSeq Standard- Each pool is denatured and diluted to 350pM with 1% PhiX control library (Illumina) added. The resulting pool is then loaded into the appropriate NovaSeq Reagent cartridge (Illumina), as determined by the number of sequencing cycles desired, and sequenced on a NovaSeq6000 instrument (Illumina) following the manufacturer's recommended protocol.

Supplementary Tables

Supplementary Table S1. List of reagents and resources used

REAGENT or RESOURCE	SOURCE	IDENTIFIER
**Antibodies (Ab)**		
Neu Ab (C-18)	SantaCruz	Cat# SC284
HSP70/HSP72 polyclonal Ab	Enzo	Cat# ADI-SPA-811-D
goat anti-rabbit IgG-HRP	SantaCruz	Cat# SC2004
Anti-β-Actin–Peroxidase Ab, Mouse monoclonal Ab	Sigma	Cat# A3854
Polyclonal Rabbit Anti-Human c-erbB-2 Oncoprotein	DAKO	Cat# A0485
HSP70 (D69) Ab	Cell Signaling	Cat# 4876
Epredia Lab Vision Ki-67, Rabbit Monoclonal Ab	ThermoFisher	Cat# RM-9106-S1
Cleaved Caspase-3 (Asp175) Ab	Cell Signaling	Cat# 9661
Survivin (71G4B7) Rabbit mAb	Cell Signaling	Cat# 2808
Actin, α-Smooth Muscle - Cy3 Ab, Mouse monoclonal Ab	Sigma	Cat# C6198
Anti-LINE-1 ORF1p Ab, clone 4H1	Sigma	Cat# MABS1152
NF-κB p65 (D14E12) XP Rabbit mAb	Cell Signaling	Cat# 8242
Donkey Anti-Mouse IgG Ab, Cy3 conjugate	Sigma	Cat# AP192C
Donkey anti-Mouse IgG (H+L) ReadyProbes Secondary Ab, Alexa Fluor 488	ThermoFisher	Cat# R37114
Polyclonal Rabbit Anti-mouse-LINE-1 ORF1p antibodies	GenScript	In this study
**Chemicals, kits, supplies**		
Stavudine (STV)	Synthonix	Cat# S2693
17-dimethylaminoethylamino-17-demethoxygeldanamycin (17-DMAG)	MedChemExpress	Cat# HY-12024\CS-0162
DMEM F-12	Corning	Cat# 10-013-CV
Fetal Bovine Serum	Gibco	Cat# 10437-028
Pen Strep	Gibco	Cat# 15140-122
GenJet Plus reagent	SignaGen	Cat# SL100499
Blasticidin S	ThermoFisher	Cat# BP264725
Doxorubicin	Sigma	Cat# D1515
Methanol	Alfa Aesar	Cat# 31721
Methylene blue dye	USB	Cat# 19220
Sodium dodecyl sulfate (SDS)	ThermoFisher	Cat# 28312
PBS buffer	Corning	Cat# 21-031-CV
Doxycycline	Sigma	Cat# D3072
Coelenterazine	GoldBio	Cat# CZ2.5
Sodium ascorbate	Sigma	Cat# A7631
Triton X-100	Sigma	Cat# X-100
Trizol	ThermoFisher	Cat# 15596018
DNAse I	Sigma	Cat# 11284932001
iTaq Universal One-Step Kit	BioRad	Cat# 1725150
Non-fat dry milk	RPI	Cat# M17200
Tris base	ThermoFisher	Cat# BP152-5
Sodium chloride	ThermoFisher	Cat# BP358-10
Tween-20	Sigma	Cat# P1379
RIPA buffer	Sigma	Cat# 20-180
Quick start Bradford reagent	BioRad	Cat# 500-0205
Bovine serum albumine	Sigma	Cat# A4503
Precision Plus Protein Dual Color Standards	Bio-Rad	Cat# 1610374
Immun-Blot PVDF membrane	Bio-Rad	Cat# 1620177
Western Lightning Plus, Chemiluminescent Substrate	Perkin Elmer	Cat# NEL105001
R-Universal antigen retrieval buffer	Electron Microscopy Sciences	Cat# 62719-10

Donkey serum	Sigma	Cat# D9663
Hematoxylin	Sigma	Cat# H9627
Ultravision ONE HRP polymer kit	ThermoFisher	Cat# TL-060-HLJ
Fisher Chemical Permount Mounting Medium	ThermoFisher	Cat# SP15-100
Formaldehyde	PolySciences	Cat# 04018
Glycine	Sigma	Cat# G7126
DAPI	ThermoFisher	Cat# D1306
ProLong Gold antifade reagent	ThermoFisher	Cat# P36930
miRNeasy mini kit	Qiagen	Cat# 217084
Qubit Broad Range RNA kit	ThermoFisher	Cat# Q10210
RNA Nanotape	Agilent Technologies	Cat# 5067-5576
RNA HyperPrep Kit with RiboErase (HMR) kit	Roche Sequencing Solutions	Cat# KK8560
KAPA Pure beads	Roche Sequencing Solutions	Cat# KK8000
KAPA Biosystems qPCR kit	Sigma	Cat# KR0397
PhiX control library	Illumina	Cat# FC-110-3001
NovaSeq Reagent cartridge	Illumina	Cat# 20028312
**Experimental models: Cell lines**		
4T1	ATTC	Cat# CRL-2539
**Experimental models: Organisms/strains**		
FVB/N-TgN (MMTV-HER2/neu) 202Mul/J	Jackson Laboratory	Strain #002376
129X1/SvJ-Tg(TH-MYCN)41Waw/Nci	NCI Mouse Repository	Gift of William Weiss (USCF, CA)
**Recombinant DNA**		
Super PiggyBac Transposase Expression Vector	System Biosciences	Cat# PB21OPA-1
XLone-GFP plasmid	Addgene	Cat# 96930
GLuc-based retrotransposition readout cassette (GLucAI)	GenScript	in this study
pTRE3G-BI-Luc Control plasmid	Takara Bio	N/A
Tet-On® 3G Bidirectional Inducible Expression System (EF1alpha Version)	Takara Bio	Cat# 631340
**Software and algorithms**		
ZEN software 2.6 version	Zeiss	N/A
FastQC	Babraham Bioinformatics	N/A
STAR RNA-seq aligner	Encodeproject	N/A
featureCounts	Bioconductor	N/A
DESeq2	Bioconductor	N/A
R clusterProfiler package	Bioconductor	N/A
TEtranscripts	Hammel Lab	N/A
SigmaPlot 11	SigmaPlot	N/A
GraphPad Prism 9 software.	GraphPad Software Inc.	N/A
**Equipment**		
Perkin Elmer Victor 3x plate reader	Perkin Elmer	N/A
BioRad CFX 384 machine	BioRad	Cat# 1855484
Beckman tabletop centrifuge	Beckman	N/A
ChemiDoc MP Imaging System	BioRad	Cat# 12003154
Zeiss AxioImager 2 microscope	Zeiss	N/A
AxioCam 512 digital camera	Zeiss	N/A
AxioCam 702 digital camera	Zeiss	N/A
4200 Tapestation	Agilent Technologies	G2991BA
NovaSeq6000 instrument	Illumina	N/A

Supplementary Table S2. List of PCR primer sequences

Primer name (gene/direction)	Primer sequence 5'-3'
mGAPDH Fwd	GGCAAGTTCAACGGCACAGTCAAG
mGAPDH Rev	GCACATACTCAGCACCAGCATCAC
mNFKBIA Fwd	TGCAGGCCACCAACTACAAT
mNFKBIA Rev	GAGCGAAACCAGGTCAGGAT
mFAS Fwd	GTCCTGCCTCTGGTGCTTG
mFAS Rev	AGCAAAATGGGCCTCCTTGA
mBCL3 Fwd	CAACATCTACACCTGCCTTCC
mBCL3 Rev	GAAGCAGAACAAGGTCCTCAC
mNFKB1 Fwd	GTCAAAATTTGCAACTATGTGGGG
mNFKB1 Rev	GTTTGCAAAGCCAACCACCA
mIRF1 Fwd	TGTCGTCAGCAGCAGTCTCTC
mIRF1 Rev	TTCGGCTATCTTCCCTTCCTC
mIL1b Fwd	GCCACCTTTTGACAGTGATGAG
mIL1b Rev	AGCTTCTCCACAGCCACAAT
mIL15 Fwd	TGCAGTGCATCTCCTTACGC
mIL15 Rev	AGGTGGATTCTTTCCTGACCTC
mHSPA8 Fwd	GTCACAGTGCCCGCTTACTT
mHSPA8 Rev	TCAAAAGTGCCACCTCCCAA
mHSPA9 Fwd	TCCAGCCACCATGATAAGCG
mHSPA9 Rev	TACCAACCACTGCACCCTTG
mHSP90AA1Fwd	AGATCCCCAGACCCATGCTA
mHSP90AA1Rev	CTTCCATGCGTGATGTGTCG
mL1 Fwd	TGGCTTGTGCTGTAAGATCG
mL1 Rev	TCTGTTGGTGGTCTTTTTGTC
mSINE1 Fwd	GAGCACACCCATGCACATAC
mSINE1 Rev	AAAGGCATGCACCTCTACCACC
mERV Fwd	TAGATGGAGCCTACCAAGCTCTCAA
mERV Rev	AGAGGTATGGTTGGAATAAGTA
